# Examination of the Public’s Reaction on Twitter to the Over-Turning of Roe v Wade and Abortion Bans

**DOI:** 10.3390/healthcare10122390

**Published:** 2022-11-29

**Authors:** Heran Mane, Xiaohe Yue, Weijun Yu, Amara Channell Doig, Hanxue Wei, Nataly Delcid, Afia-Grace Harris, Thu T. Nguyen, Quynh C. Nguyen

**Affiliations:** 1 Department of Epidemiology & Biostatistics, University of Maryland School of Public Health, College Park, MD 20742, USA; 2 Department of Behavioral and Community Health, University of Maryland School of Public Health, College Park, MD 20742, USA; 3 Department of City and Regional Planning, Cornell University, 921 University Ave, Ithaca, NY 14850, USA; 4 McKinney Public High School, 1400 Wilson Creek Pkwy, McKinney, TX 75069, USA; 5 Department of Public Health Science, University of Maryland School of Public Health, College Park, MD 20742, USA

**Keywords:** Roe v Wade, abortion, women’s rights, pro-choice, pro-life, family planning, sentiment analysis, network analysis

## Abstract

The overturning of Roe v Wade reinvigorated the national debate on abortion. We used Twitter data to examine temporal, geographical and sentiment patterns in the public’s reaction. Using the Twitter API for Academic Research, a random sample of publicly available tweets was collected from 1 May–15 July in 2021 and 2022. Tweets were filtered based on keywords relating to Roe v Wade and abortion (227,161 tweets in 2021 and 504,803 tweets in 2022). These tweets were tagged for sentiment, tracked by state, and indexed over time. Time plots reveal low levels of conversations on these topics until the leaked Supreme Court opinion in early May 2022. Unlike pro-choice tweets which declined, pro-life conversations continued with renewed interest throughout May and increased again following the official overturning of Roe v Wade. Conversations were less prevalent in some these states had abortion trigger laws (Wyoming, North Dakota, South Dakota, Texas, Louisiana, and Mississippi). Collapsing across topic categories, 2022 tweets were more negative and less neutral and positive compared to 2021 tweets. In network analysis, tweets mentioning woman/women, supreme court, and abortion spread faster and reached to more Twitter users than those mentioning Roe Wade and Scotus. Twitter data can provide real-time insights into the experiences and perceptions of people across the United States, which can be used to inform healthcare policies and decision-making.

## 1. Introduction

**Figure A1:**
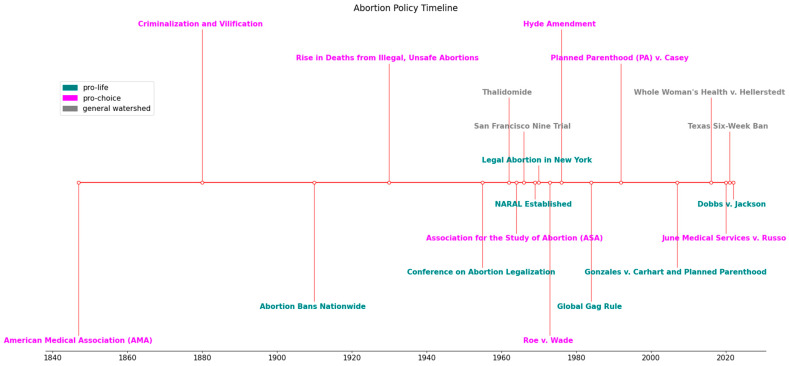
Abortion Policy Timeline.

The long and controversial debate about abortion did not begin with Roe v Wade. Prior to the early 1800s, abortion was legal and widely practiced in the United States. By some estimates, the mid-19th century saw up to 25% of pregnancies end in abortion [1]. The criminalization of abortion steadily proliferated across the United States throughout the 19th century. By 1900, all U.S. states had adopted laws prohibiting abortion and marking the start of an era of unsafe and illegal abortions. In 1930, the Guttmacher Institute estimated that illegal and unsafe abortions accounted for up to 18% of recorded maternal mortality for that year [2]. In 1955, the alarming increase in unsafe abortions prompted a Conference on Abortion Legalization where medical professionals advocated for abortion law reform. In 1962, an increase in birth defects associated with Thalidomide, a drug used by women used to ease pregnancy symptoms, lead to further advocacy for reforming abortion-restricting laws. For additional information on the timeline of relevant events related to Roe v Wade leading up to the overturn refer to Figure A1 in the Appendix A. On 22 January 1973, the Supreme Court issued its ruling on Roe v Wade, striking down abortion restrictions in Texas and legalizing abortion as a constitutionally protected procedure. Roe v Wade has proven to be an enduring touchstone for U.S. public discourse and debate over the nation’s values and jurisprudence, splitting the nation into two camps: pro-life groups that oppose abortion access and pro-choice groups that support abortion access. In recent years, the cultural conflict over abortion has spread and expressed itself in public protests, letter-writing campaigns, op-eds in Sunday papers, and even violence.

In Roe v Wade, a majority of the Supreme Court justices ruled that the 14th Amendment contained a right to privacy, which provided for a woman’s right to an abortion. The majority opinion scoped the right to privacy as “broad enough to encompass a woman’s decision whether or not to terminate her pregnancy” [3]. Under the umbrella of a right to privacy, the Roe v Wade ruling jettisoned religious and moral considerations on the legality of abortion in favor of health and wellness, specifically emotional, physical, and psychological health [4]. Categorizing abortions as a medical decision made the procedure available to women regardless of their reasons, which have included life-threatening complications, contraceptive failure, financial burdens, rape, or intimate partner violence. According to the CDC, 614,820 abortions were reported in the United States in 2018. The abortion rate was 11.3 abortions per 1000 women aged 15 to 44. The abortion ratio was 189 abortions per 1000 live births. Among the age group measured by the CDC, women in their twenties account for 57.7% of abortions in 2018 [5].

The right-to-privacy precedent would provide the foundation for future rulings expanding individual rights, including, e.g., Planned Parenthood v Casey (1992), which permitted states to regulate abortion services before viability as long as it does not place undue burden on women seeking an abortion. On 3 May 2022, Supreme Court Justice Samuel Alito’s draft opinion for the 6 to 3 majority in Dobbs v. Jackson Women’s Health Organization Supreme Court case was leaked, reinvigorating the national debate on the right to have an abortion. Nearly 50 years after its initial ruling, Roe v Wade was overturned on 24 June 2022, by the Dobbs v. Jackson case, which also overruled Planned Parenthood v. Casey. The Dobbs v. Jackson case ruled that a Mississippi law banning abortions after 15 weeks was constitutional. The law was designed to provide a legal basis by which to overturn the Roe v Wade ruling, which states that abortion cannot be banned before the fetus reaches viability (24–28 weeks into pregnancy). The ruling did not ban abortion; it removed the constitutional restriction on legislation banning abortion.

The legal ramifications of the Dobbs v. Jackson have largely occurred at the state level. State trigger laws and fast-acting state [5] legislatures have instituted near-total bans, six-week bans, and pending bans. Near total bans on abortion have been instituted in Alabama, Arkansas, Kentucky, Mississippi, Missouri, Oklahoma, South Dakota, and Wisconsin; six-week abortion bans are now law in Georgia, Ohio, South Carolina, Tennessee, and Texas; and bans are pending in Idaho, North Dakota, and Wyoming. Since the ruling, there have been have reports of delay of care, for instance in ectopic pregnancies and miscarriages, which increase the risk of maternal morbidity and mortality [6,7]. This overturn may especially affect socioeconomically vulnerable groups who have worse healthcare access and are less able to take time off work and travel out-of-state as necessary to have abortions. Providing access to safe and accessible abortion care can prevent adverse side effects that women may face due to unsafe abortions [8,9].

Dobbs v. Jackson and subsequent state-level abortion bans have evoked strong reactions from “pro-life” and “pro-choice” groups [4]. Many studies on attitudes towards Roe v Wade have used surveys [10,11]. However, much of the public reaction to Dobbs v. Jackson has been expressed on social media. As an interactive web-based technology, social media has connected the world and has facilitated the generation and sharing of information, ideas, and sentiments on a range of topics, including abortion. Many public health researchers have recognized and seized the unique opportunity to survey and collect social media data in real time to understand changes in public attitudes and sentiment [12,13]. Twitter, a popular microblogging social media platform, has been widely used as a resource for studying public opinion and sentiment around various topics [14,15], self-reported health behaviors [15], the consumption and dissemination of information [16], and has been an effective tool for health and disease surveillance [17]. It offers up-to-the-minute insight into consequential societal happenings and the broader discourse surrounding those happenings. Previous research examined whether killings of George Floyd, Ahmaud Arbery, and Breonna Taylor by current or former law enforcement officers in 2020 were followed by shifts in public sentiment toward Black people [18]. Other work exploring U.S. Shifts in Anti-Asian Sentiment with the emergence of COVID-19 [19]. Building on the existing body of work on social media and public health, we examine temporal and geographical patterns in the public’s reaction to the overturning of Roe v Wade.

Studying public health topics at a population level through traditional data collection methods can be time-consuming and expensive. Social media platforms such as Twitter provide efficient and cost-effective data sources that enable researchers to evaluate sensitive and real-time health topics in a large population [20]. Social media has been ubiquitous in sharing and consuming information, and researchers have been observing that it has also been pervasive in the healthcare landscape. More users are using social media to learn information, share reviews of healthcare facilities and physicians, and share their opinions and experiences [21]. A past study used social media to examine abortion attitudes among adolescents and young adults in the United States [22]. Another study examined Twitter messaging strategies used by anti- and pro-abortion organizations in Ireland [23]. Our study addresses the knowledge gap on using social media to examine the overturning of Roe v Wade in the United States, to enable future studies that seek to trace how Twitter discussions and topics anticipate and correlate with women’s access to reproductive healthcare.

## 2. Study Aims and Hypotheses

In this study, we used Twitter data to examine temporal, geographical and sentiment patterns in the public’s reaction to the overturning of Roe v Wade. Our hypotheses are:

**Hypothesis 1** **(H1).** *Following the leak of the Supreme Court opinion and the overturning of Roe v Wade would cause an increase in Twitter conversations about Roe v Wade and abortion-related topics, and that conversations would be most abundant in states with trigger abortion bans if Roe is overturned.*

**Hypothesis 2** **(H2).** *Compared to the pre-period, sentiment of tweets following the overturning of Roe v Wade would be more positive for women’s rights, family planning, and abortion.*

**Hypothesis 3** **(H3).** *Tweets referencing abortion and the Roe v Wade case would be more negative, given the controversy and disagreements across sides.*

**Hypothesis 4** **(H4).** *Tweets with a personalized component would have greater user engagement and reach a wider audience.*

### 2.1. Data Collection

Data was collected through Twitter API for Academic Research. A random 1% sample of publicly available tweets was collected from two time periods: (1) 1 May 2021, through 15 July 2021—a year before the overturning of Roe v Wade (control period), and (2) 1 May 2022, through 15 July 2022—after the leak of U.S. Supreme Court opinion and the overturning of Roe v Wade (event period). There were approximately 30 million (29,759,177) tweets in the control period and 31 million (31,038,666) tweets in the event period. The Twitter API endpoints allow us to select a set of parameters, such as user name, profile, and tweet location and receive just the data we need. Therefore, we restricted our analyses to English-language tweets from only the U.S. and for further detailed geographic analysis, we sent API requests with location related parameters and collected corresponding information, including tweets latitude and longitude coordinates (if available), place id and full names, bounding boxes, user profile location, place type or other “place” attributes that permitted identification of the U.S. region from which the tweet was sent or the user lived. Given the very low number of tweets with exact geocoordinates with where the tweet was sent, we also used the user profile location which is provided by the Twitter API. When setting their profile, users can specify their profile location and we used this information to assign tweets to a state location. Relevant event related tweets were identified based on the use of one or more keywords (e.g., abortion) or key phrases (e.g., family planning) relating to the supreme court, the Roe v Wade decision, abortion, contraceptives, women’s rights, pro-life, and pro-choice among other topics (full keyword list is in Table A1 in the Appendix A). The keywords and key phrases were identified through a literature search of words pertaining to abortion and as well as topics that emerged in popular press following the overturning of Roe v Wade. These include press coverage of concern about women’s safety, reproductive rights, and family planning as abortion became less of an option in some states. The total number of event-related tweets was 227,161 for the control period and 502,117 for the event period. There were 137,921 unique users from the control period and 116,677 unique users from the event period.

### 2.2. Analytic Approach

Based upon the keywords and key phrases, categories were created by grouping synonyms or similar words such that frequency of topic categories could be quantified (Table A2 in the Appendix A). This was because some specific keywords had lower frequency than others but when combined as a group of, we can better estimate the popularity of that topic. Roe v Wade-related terms were grouped into the following eight categories: (1) pro-life, (2) pro-choice, (3) female/women, (4) abortion, (5) contraceptives, (6) women’s rights, (7) roe v wade, (8) other. The frequency of Roe v Wade-related categories was calculated and plotted against time to show temporal trends. Additionally, we conducted sentiment analysis to examine changes in sentiment for these categories across event and control periods. We also retrieved a random sample of 1000 tweets from each of the seven keyword categories. Example tweets in each category were selected and displayed in Table 1 to give readers a sense of the content of the tweets.

### 2.3. Sentiment Analysis

For sentiment classification, we used Support Vector Machine (SVM), a supervised machine learning algorithm that is used for text classification in natural language processing tasks. Prior to classification, the data was cleaned and processed. Duplicated tweets were identified based on repeated “tweet_ids” and were dropped. Stopwords which are terms commonly used that hold no analytical significance such as “in”, “the”, and “that”, numerical characters, punctuations, emojis, URLs, and links were also removed from the corpus of tweets. To train the SVM classification model, we annotated 6481 tweets for the sentiment. For each tweet, we created two dichotomous sentiment labels—negative (yes/no) and positive (yes/no). Tweets that did not fall in negative or positive were labeled neutral. Additional details of this method have been published elsewhere [18]. We also obtained publicly available sentiment-labeled data from Sentiment140 (*n* = 498) [24], Kaggle (*n* = 7086) [25], Sanders (*n*= 5113) [26]. After training the SVM, to test the effectiveness of the machine learning algorithm in classifying sentiment, we used the 5-fold cross-validation method. Our trained model yielded an F1 score of 84% and an accuracy rate of 91%. In other words, the model predictions matched the manual-labeled sentiments 91% of the time. Sample tweets with their sentiment categorization are included in the Appendix A Table A3. T-tests compared changes in sentiment between 2021 and 2022. We applied the Bonferroni correction by dividing the critical *p* value (α) by the number of comparisons being made (8 × 3 = 24 overall comparisons). This gives us a revised alpha level of 0.002.

### 2.4. Network Analysis

We built an undirected network [27] for hubs [28] composed of tweets from the event period with a large number of likes (>10,000), which was used to visualize how high-impact tweets were interconnected. Tweets with less than 10,000 likes were excluded from the network analysis. Vertices represent the individual hubs while edges [29] correspond to Roe v Wade-related terms. If two vertices mentioned the same Roe v Wade-related term (i.e., abortion), an edge was created to connect the two vertices. A total of 145 vertices met the inclusion criteria and 1954 edges were created. We measured the network by computing degree, density, clustering coefficient [27], and average path length [30]. For network visualization, we used the Fruchterman Reingold algorithm for layout and weighted the size of the vertex by number of likes (increasing the number of likes indicates larger size of vertex). The U.S. geographical regions (i.e., Northeast, Midwest, South, or West) and sentiment (i.e., happy, neutral, or sad sentiment) were treated as vertex attributes and were color-coded. Network analysis was conducted utilizing the igraph package in R Statistical Software version 4.1.3. Network visualization was performed by Gephi [31].

## 3. Results

**Figure 1:**
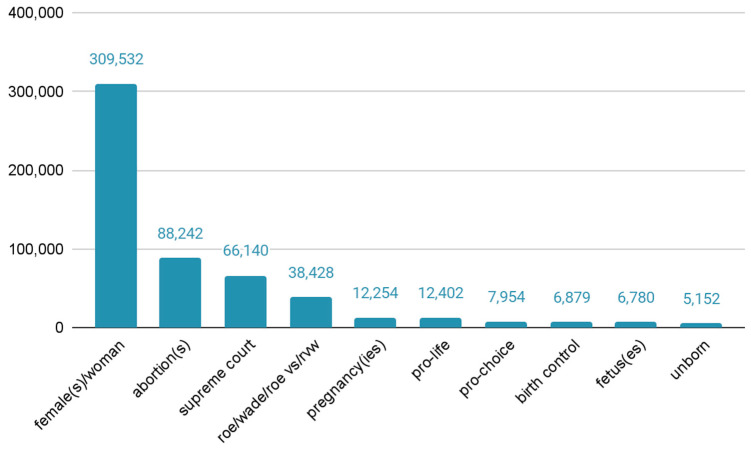
Top terms by frequency of tweet mentions, 1 May–15 July 2022.

Overall, more than 500,000 tweets were identified as containing at least one of the identified keywords. Figure 1 displays the most popular keywords during the 1 May–15 July 2022 time period. The most popular term was female/woman/girls followed by abortion(s), supreme court/scotus, roe v wade. Other less common keywords reflected discussion regarding different sides of the debate and concern over future access to birth control and emergency contraceptives.

### 3.1. Content Assessment on Example Tweets

We found that the keywords were applied in varying ways and that they were present in tweets arguing on both sides of the abortion debate. Tweets that included the phrase pro-life ranged from celebrations of the decision to highlights of religious convictions, while others criticized other conservative policies for failing to extend a pro-life ethos to policy areas beyond abortion (e.g., support for safety nets, social programs), and disparaged the pro-life movement. Tweets that included the phrase pro-choice similarly contained topics around religious convictions. They also included posts expressing anger over the decision and political discussions about electing more pro-choice politicians. Keywords categorized under abortion were similarly used in tweets that express support for the decision as well as fear and disappointment. Family planning tweets focused on using contraception to prevent the need for abortion, concerns about “abortion pills,” fear around future lack of access to birth control, and plans for long-acting contraceptive methods. Tweets that included Roe v Wade were used to celebrate or share dismay about the decision and to discuss future political needs for both conservative and liberal policies. Women’s rights and women’s tweets included concerns about the overturn of Roe v Wade having a domino effect on other human rights (i.e., basic human rights or LGBTQI+), anger with specific politicians, discussions of cases of assault, and disparaging comments about women. Example tweets for each keyword are presented in Table 1.

### 3.2. Geographic Variation in Twitter Conversations around Roe v Wade

**Figure 2:**
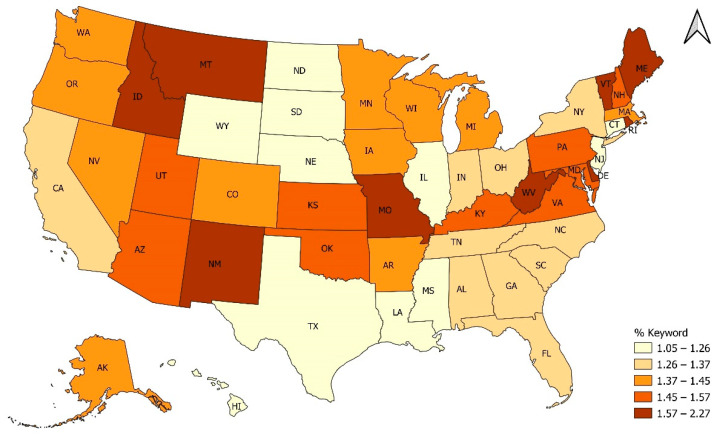
Twitter conversations about Roe v Wade as a percentage of all tweets sent 1 May–15 July 2022 by state.

**Figure A2:**
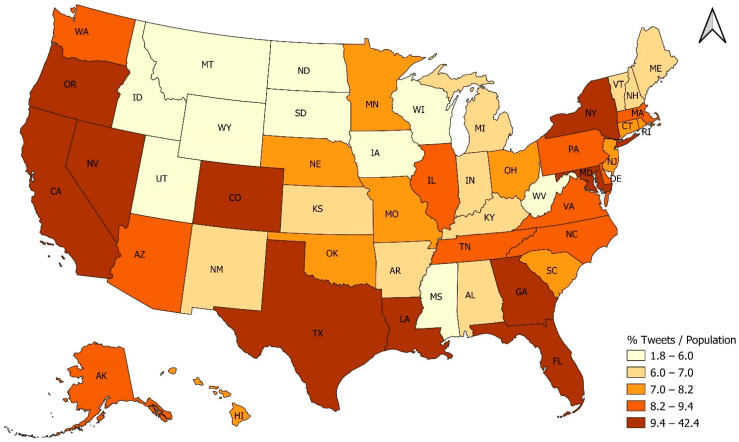
Number of tweets Roe v Wade sent 1 May–15 July 2022, divided by state population.

Figure 2 maps state-level percentages of tweets that mentioned one of more Roe v Wade keyword or phrases out of all tweets sent from a given state during 1 May–15 July 2022. Darker colors represent higher proportion of Twitter conversations about Roe v Wade and abortion-related topics. Montana, Idaho, New Mexico, Missouri, West Virginia, Vermont and Maine had the most conversations about these topics as a proportion of all tweets sent during 1 May–15 July 2022. Additionally, Utah, Arizona, Kansas, Oklahoma, Kentucky, Virginia, Pennsylvania, Maryland, and New Hampshire also saw moderately high prevalence of these conversations. States that had relatively few Twitter conversations around Roe v Wade and abortion-related topics included Wyoming, North Dakota, South Dakota, Nebraska, Texas, Louisiana, Mississippi, Illinois, New Jersey, and Connecticut. We also include a state-level map accounting for state population size in the appendix (Figure A2). Nonetheless, we believe that dividing by the total number of tweets (Figure 2) is better for determining the popularity of a Twitter topic because it also accounts for the differential number of tweets and differential Twitter use across states. 

**Figure 3:**
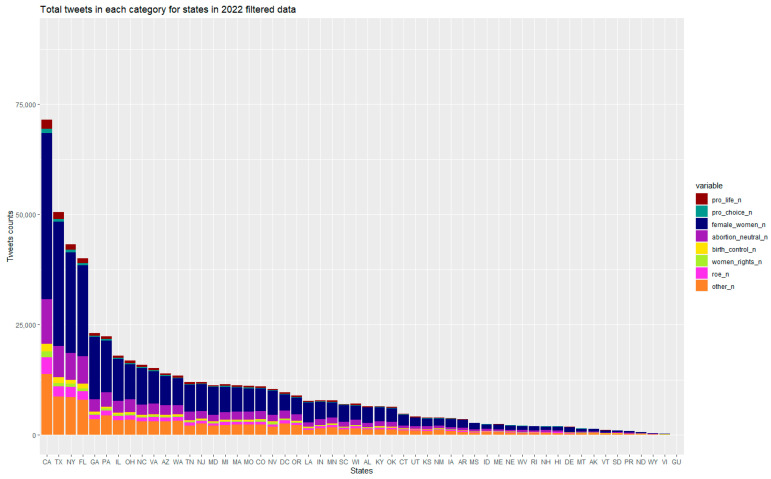
Total number of tweets in each topic category from the event-related tweets, by state (1 May–15 July 2022).

Figure 3 presents the raw quantity of Roe v Wade tweets sent from each state. States with large populations and large number of Twitter users dominate such that California, New York, Texas, Florida, and Georgia are among states with the largest number of tweets about Roe v Wade, not accounting for population size or total number of tweets.

States also differed with regard to the proportion of Twitter conversations on particular topics (Table A4 in the Appendix A). For example, states and U.S. territories with the lowest proportion of conversations on abortion included Delaware, Georgia, Louisiana, and the Virgin Islands. Delaware, Georgia, Mississippi, and the Virgin Islands also had the lowest proportion of conversations on women’s rights.

### 3.3. Comparison of May–July 2022 to May–July 2021

**Figure 4:**
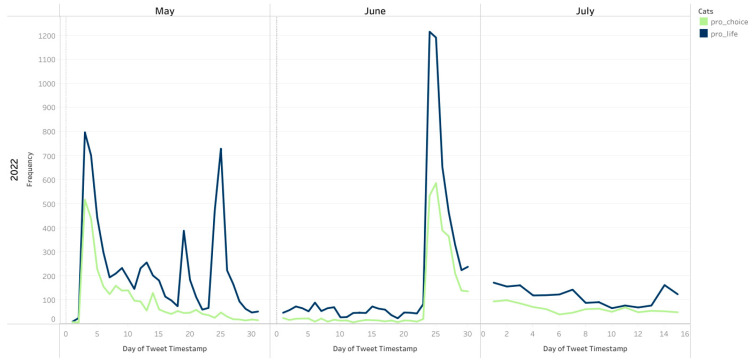
Number of pro-choice and pro-life tweets across 1 May–15 July 2022.

**Figure A3:**
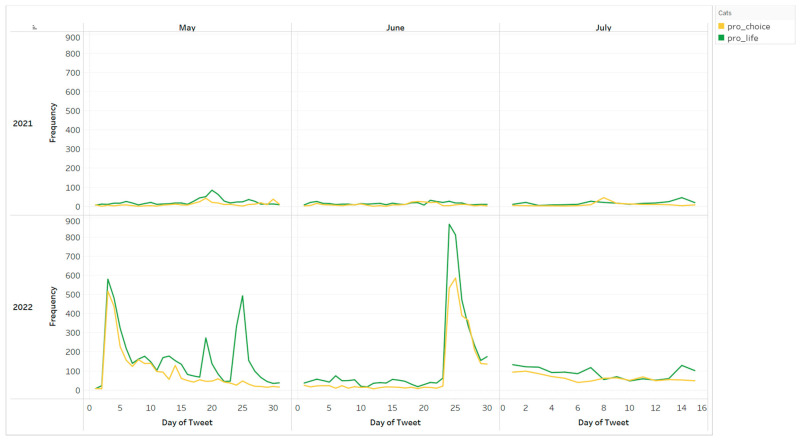
Number of pro-choice and pro-life tweets across 1 May–15 July 2021 and 2022.

**Figure A4:**
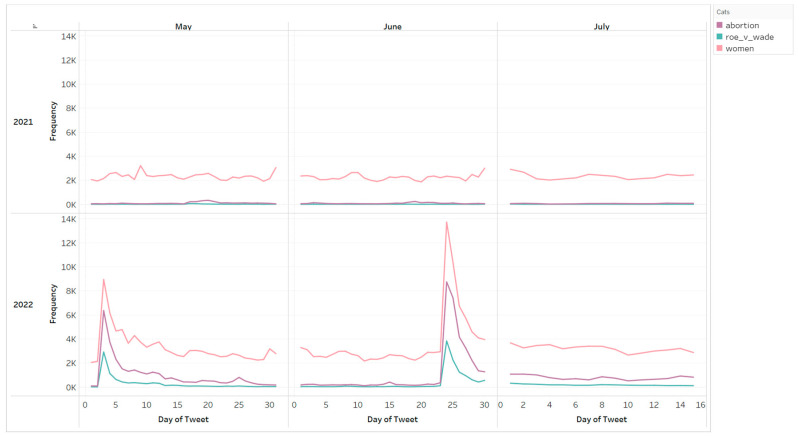
Number of Tweets about Abortion, Roe v Wade, and Women from 1 May–15 July in 2021 and 2022.

**Figure A5:**
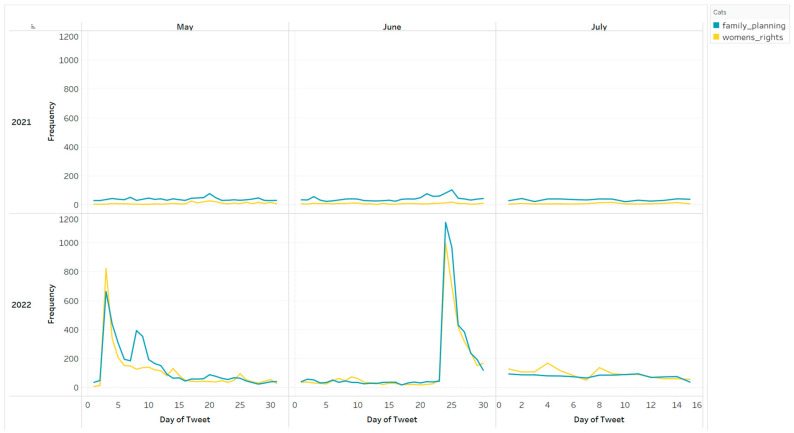
Number of tweets about Women’s Rights and Family Planning across 1 May–15 July 2021 and 2022.

Please refer to Figure A3, Figure A4 and Figure A5 in the Appendix A for year-over-year comparisons of the control period versus the treatment period. For instance, Figure A3 in the Appendix A provides a time plot of pro-choice and pro-life tweets across 1 May–15 July 2021 (control period) and 1 May–15 July 2022 (event period). The control period saw a relatively constant and low number of pro-choice and pro-life tweets. The leak to Politico of an initial draft of a Supreme Court opinion overruling Roe v Wade and Planned Parenthood v Casey occurred on 2 May 2022. The time plot reveals a spike in Twitter conversations with both pro-choice and pro-life terms during the first week of May 2022 (Figure 4). Thereafter, pro-choice tweets declined in May but pro-life conversations continued with several spikes showing renewed interest throughout May. Pro-life and pro-life conversations returned to low levels in June and then spiked on 24 June 2022, when the U.S. Supreme Court officially ruled to overturn Roe v Wade. However, during the initial few days between 24 June and 26 June, pro-life tweets were more prevalent than pro-choice tweets. The frequency of Roe v Wade conversations tapered off and flat-lined after one week (Figure 4).

**Figure 5:**
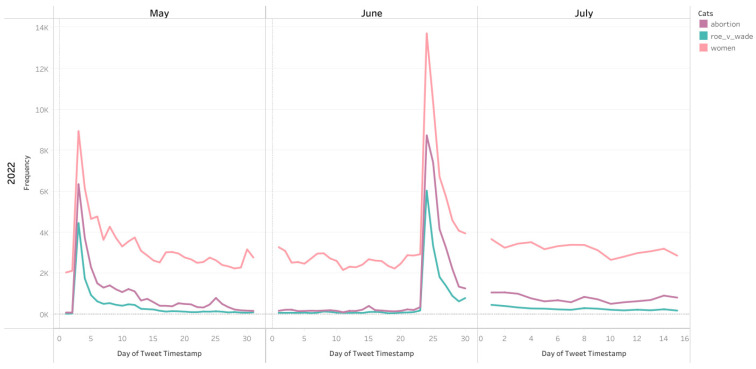
Number of Tweets about Abortion, Roe v Wade, and Women from 1 May–15 July 2022.

**Figure 6:**
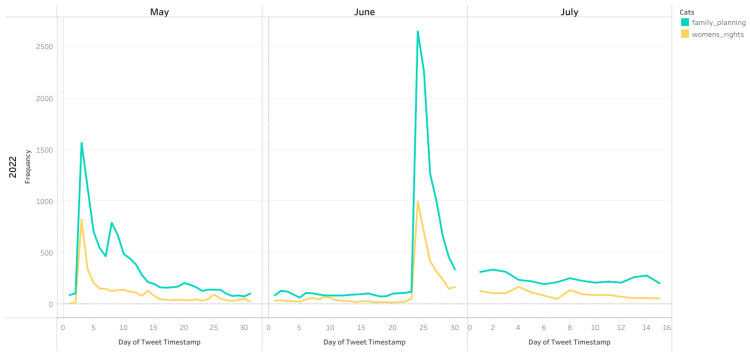
Number of tweets about Women’s Rights and Family Planning across 1 May–15 July 2022.

Similar spikes in temporal trends of other topic categories were also observed (Figure 5 and Figure 6). There was a general trend of a smaller spike in conversations after the leaked Supreme Court opinion in early May followed by a larger spike in conversations following the overturning of Roe v Wade on 24 June 2022. However, the differential between the size of the spike in conversation after the leak compared to the official overturning was largest for family planning and women which saw a much larger increase in tweets after the official overturning of Roe v Wade (Figure 6), potentially signaling much more attention to these topics following the official ruling.

**Figure 7:**
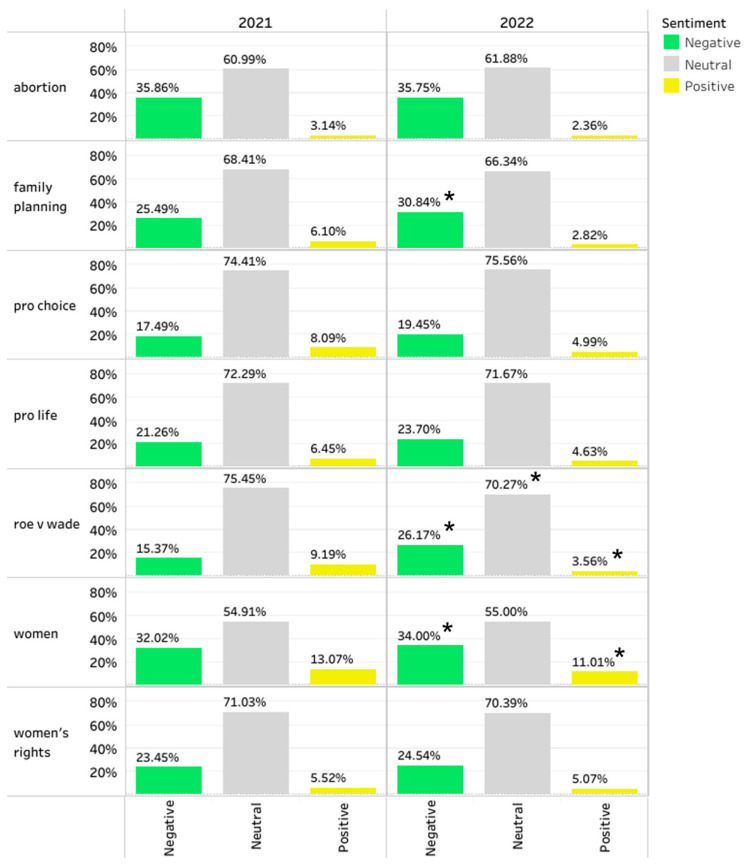
Topic categories by sentiment, 1 May–15 July in 2021 and 2022. * *p* < 0.002 for Bonferroni corrected t-tests comparing 2021 vs. 2022 sentiment.

Each tweet was assigned a sentiment label of positive, neutral, or negative. Tweets were grouped into topic categories. Figure 7 displays topic categories and their sentiment patterns. We see that across categories, neutral tweets were dominant followed by sad and then happy tweets. However, the exact proportions of these sentiment labels varied across topic categories. Family planning, pro-choice, pro-life, and roe v wade categories had the highest proportion of neutral tweets. Abortion and women topics had the highest proportion of negative tweets. Compared to 2021, 2022 saw a much greater abundance of tweets related to these topics and saw a lower proportion of neutral and positive tweets for these topics.

**Figure A6:**
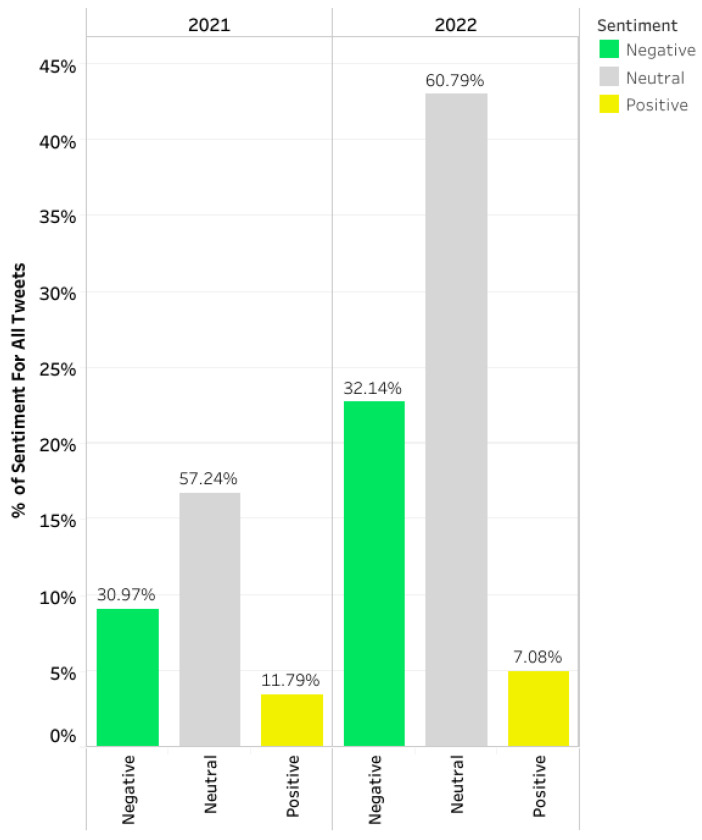
Sentiment among all keyword filtered tweets, 1 May–15 July 2021 vs. 2022. Percent changes in sentiment categories (negative, neutral, positive) between 2021 and 2022 were all statistically significant with *p* < 0.002.

*T*-tests comparing average 2021 sentiment values to 2022 sentiment values revealed statistically significant differences for family planning (higher percentages of negative sentiment), Roe v Wade (higher percentages of negative sentiment, lower percentages of positive sentiment), women (higher percentages of negative sentiment, lower percentages of positive sentiment) (Figure 7). Collapsing across topic categories, 2022 tweets were more negative and less neutral and positive compared to 2021 tweets (Figure A6 in the Appendix A).

**Figure 8:**
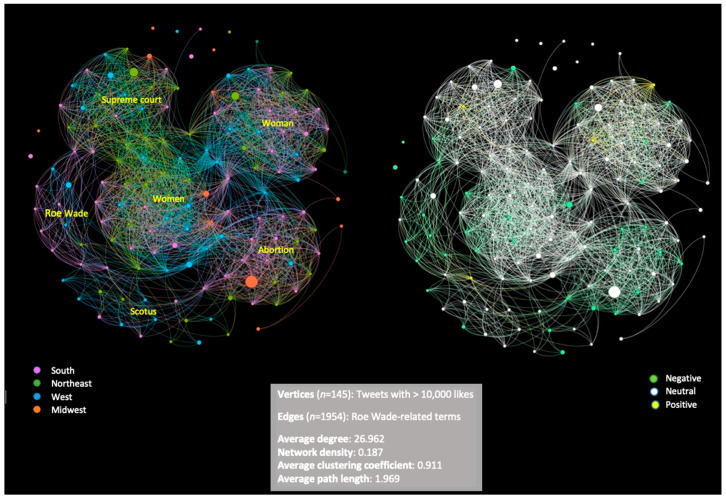
Network of Roe Wade-related Tweet Hubs by U.S. regions and Sentiment (May–July 2022).

Figure 8 displays the network topology of our tweet hubs. The subfigure on the left shows the network layout color-coded by four geographical regions whereas the subfigure on the right was color-coded for happy, sad, and neutral sentiments. Among the 145 tweet hubs, we detected four densely connected clusters for Roe v Wade-related terms: (1) women, (2) woman, (3) supreme court, (4) abortion, and two sparsely connected clusters: (1) Roe v Wade, (2) SCOTUS. This indicates that tweets mentioning woman/women, the supreme court, and abortion spread faster and reached more Twitter users than those mentioning Roe v Wade and SCOTUS. Three super-hubs (vertices with the biggest sizes and most likes) with neutral sentiment were found: abortion from Midwest, and supreme court and women from the Northeast. Tweet hubs from the Northeast are more likely to mention women in the plural form while those from the South tend to mention ‘woman’ in the singular form. This may signal potential geographical and cultural differences in tendencies for individualistic vs. collectivist framing of issues. Example tweet from the Northeast states include, “we need to come together as one regardless of color and social status pray that women can look at today as setback yrs for all women” and “Guns have more rights than women in America.” Most tweet hubs regarding abortion and Roe v Wade show negative sentiment and are from the South. The rest of the clusters indicate neutral sentiment. Tweet hubs from the Northeast focus on the supreme court and women, while those from the West cover all the related terms sporadically. The Midwest has the least number of tweet hubs. Tweet hubs with positive sentiment are very few and observed in clusters of woman and supreme court. For network measures, average degree of 26.96 indicates that one tweet hub on average in our network shares the same Roe v Wade-related term with approximately twenty-seven tweet hubs. Network density 0.187 shows that our network is similar to many real-life networks, which is a sparse network. Average path length of 1.969 and average clustering coefficient 0.911 imply that our network is well connected.

## 4. Discussion

In alignment with our study hypotheses and with prior research examining major events, we did observe a high increase in Twitter conversation about Roe v Wade and abortion-related topics following the Supreme Court opinion leak and the official overturning of Roe v Wade. However, counter to study hypotheses for changes in sentiment across topic categories, uniformly across topic categories sentiment became more negative and less neutral and positive in 2022 compared to 2021, which may represent the polarizing effect of the decision. We hypothesized that states with trigger abortion bans following Roe v Wade would have the most prevalent conversations and this was not consistently observed because some states with trigger laws had fewer Roe v Wade conversations. In network analysis, tweets mentioning woman/women, supreme court, and abortion spread faster and reached to more Twitter users than those mentioning Roe Wade and Scotus, which are in line with study hypotheses that terms indicative of more personal stories would have higher user engagement.

The reversal of Roe v Wade determined that in fact, the constitution has no right to privacy, and it empowered states to decide on the legality of abortion, effectively, making geography a determinant of access to legal and safe abortion in the U.S. In alignment with prior research examining social media’s reaction to major events, across topic categories, we saw a small spike in conversations after the Supreme Court opinion leaked in early May followed by a larger spike in conversations following the overturning of Roe v Wade on 24 June. The differential between the size of the spike in conversation after the leak compared to the official overturning was largest for family planning and women which saw a much larger increase in tweets after the official overturning of Roe v Wade, potentially signaling much more attention to these topics following the official ruling. With regard to tweets containing ‘pro-life’ and ‘pro-choice’ terms, the time plots revealed a spike in Twitter conversations for both topics during the first week of May 2022. Thereafter, pro-choice tweets declined in May but pro-life conversations continued with several spikes showing renewed interest throughout May. Tweets increased again after the official overturn of Roe v Wade, with pro-life tweets were more prevalent than pro-choice tweets.

Our study additionally investigated geographical differences in Twitter engagement with Roe v Wade and abortion-related topics. We hypothesized that states with trigger abortion bans following Roe v Wade would have the most prevalent conversations and this was partially observed. States with abortion trigger bans that took place automatically or by quick state action after Roe is overturned included: Arkansas, Idaho, Kentucky, Louisiana, Mississippi, Missouri, North Dakota, Oklahoma, South Dakota, Tennessee, Texas, Utah, and Wyoming [32]. Twitter conversations about Roe v Wade and abortion-related topics were prevalent in Montana, Idaho, New Mexico, Missouri, West Virginia, Vermont and Maine. Additionally, Utah, Arizona, Kansas, Oklahoma, Kentucky, Virginia, Pennsylvania, Maryland, and New Hampshire also saw moderately high prevalence of these conversations as a percentage of all tweets sent. The lower prevalence of relevant tweets Wyoming, North Dakota, South Dakota, Texas, Louisiana, and Mississippi was incongruous to our expectations considering the reversal of Roe v Wade would more directly be felt in these states through trigger laws that either banned or threatened to ban abortion access. This unexpected discrepancy however, could be the result of cultural differences especially around how and where to discuss topics like abortion and/or could be indicative of differences in Twitter usage.

Across topic categories, neutral tweets were dominant followed by negative and then positive tweets. However, family planning, pro-choice, pro-life, and roe v wade categories had the highest proportion of neutral tweets. Abortion and women topics had the highest proportion of negative tweets. Examining changes in sentiment between 2021 and 2022, we had hypothesized that conversations would be more positive for women’s rights, family planning, and abortion and that tweets referencing abortion and the Roe v Wade case would be more negative. However, uniformly across topic categories sentiment became more negative and less neutral and positive in 2022 compared to 2021, which may represent the polarizing effect of the decision, increasing polarizing of U.S. electorate, public dissent with the decision among other factors which necessitate additional research inquires.

A previous study examined a network of 26,681 majority opinions written by the U.S. Supreme Court and the cases that cite them, and identified Roe v. Wade as one of the twenty most important legal cases in the United States [33]. Other researchers conducted a content analysis and social network analyses to examine Twitter conversations on abortion in Brazil [34]. From our network analysis, we found that tweets mentioning woman/women, the supreme court, and abortion garnered more user engagement than those mentioning Roe v Wade and Scotus. The later terms might be more related to the sharing of news stories whereas the former terms, in particular, around woman/women and abortion may reflect sharing of personal opinions and experiences which spread faster and reached more Twitter users.

Additionally, tweet hubs from the Northeast were more likely to use the plural form of “women” while those from the South were more likely to use the singular form of “woman,” which may point to differences in the conceptualization of issues as they pertain to a group (i.e., women) or to an individual (i.e., woman). To conduct the network analyses, we built a partial network that enabled us to specifically zoom into 145 hubs consisting of tweets with the most likes. Each vertex in our network represents a tweet with over 10,000 likes which potentially indicates that at least 10,000 users viewed this tweet and acknowledged or agreed with the information it delivered. As a result, our network can reveal patterns among at least 1.45 million users. We believe that examining the relationship and super-hubs from a small number of hubs in social networks is an innovative and cost-effective method to examine the public’s opinion on research topics. Social media users are highly likely to view tweets that share similar keywords or topics from their browser cookies history [35]. Thus, the relationship examined in our undirected network can explain information spread efficiency, which is another strength of this study. Information spread efficiency is usually examined in directed networks [36,37] which require a significant time investment for data collection and cannot be performed under most circumstances. Hence, we provide an innovative solution for future studies to examine how information spreads using undirected networks.

### 4.1. Study Findings in Context

A recent study found that Google searches for the term “vasectomy” reached a 5-year high period following the overturning of Roe v Wade. States with the highest search rate included Utah, Texas and North Dakota, which we found in our study had relatively fewer Twitter conversations about Roe v Wade or other abortion-related terms. Thus, it seems of that some of the states that talked least about Roe v Wade and abortion on Twitter searched most for the term “vasectomy” on Google. Additionally, the four states that had the highest Google searches for “vasectomy” also had trigger abortion laws in place in anticipation of the Roe v Wade ruling. The four states with the lowest Google searches for “vasectomy” did not have those trigger laws [38]. Google Trends data examines anonymized search histories while our analysis of Twitter data examines public posts. Among terms included in our Twitter keyword list was contraceptives and family planning, which we also saw an increase in following the Roe decision. The study cited using Google search data potentially sheds light on geographical differences in reaction to the abortion ban. States that might have more restricted abortion laws and social norms against abortion might discourage individuals from posting publically about their concerns. However, privately, they might search online for information on family planning methods.

Previous surveys examining abortion attitudes by the Pew Research Center found public’s attitudes on this issue remained stable across decades [39]. For instance, from 1995 to 2022, the proportion of Americans who believed that abortion should be legal in all or most cases held steady at around 60–62%. Studies have also shown that public attitudes towards abortion are stable both at individual and aggregated levels [40,41]. This is particularly remarkable given the demographic and attitudinal shifts in the American electorate. For example, attitudes towards sex outside marriage has become more accepted and there is greater support for gender equality. The percentage of women who self-identify as homemakers decreased from 28% in 1972 to 12% in 2000. Additionally, ‘moderate’ viewpoints towards abortion do not necessarily represent indifference but may reflect conflicts between deeply held beliefs. For instance, a substantial proportion of Americans believe that abortion is a “…decision made by a woman and her doctor” and that “abortion is murder.” The conflicting viewpoints may help explain the stable majority of Americans who support abortion, at least under specific situations.

Additionally, previous studies have found that abortion rates and access to safe and legal abortion vary across sociodemographic groups. Studies find that white women tend to have greater access than black women to preventative and ongoing healthcare that might preclude the need for abortion [42]. Studies also find that accesses to safe and legal abortion are strongly linked to gains in economic security [43,44]. This ongoing debate merits further study to understand the drivers and implications of abortion access for women of every economic status and race/ethnicity heritage. Using Twitter is one possible way to track in real-time the viewpoints and experiences of people across the country on abortion due to changing laws and shifting landscapes.

Existing literature also indicated that after the overturn of Roe Wade, telemedicine for medication abortion may provide access to those who need the abortion service. However, minoritized women of younger reproductive age with lower socioeconomic status still met barriers to access telemedicine for abortion [45]. States where abortions became illegalized simultaneously disabled residency programs that offer abortion training [46]. Increases in travel distances are predicted to prevent about 100,000 women from accessing abortion care. [47].

Although Twitter users are not nationally representative, the use of Twitter data has proved to be informative of people’s perceptions and experiences and has been used to provide a way to characterize different social and cultural environments. For example, Twitter data has been used to characterize food and exercise culture across the United States and those characteristics were related to varying chronic conditions [48,49]. Twitter has also been used to characterize racial sentiment hostility and linking those to adverse birth outcomes [50]. Twitter is increasingly being used to gauge public sentiment around societal issues and events. Previous studies have used Twitter to assess public sentiment in real time and found it to match with more established methods. One study looked at a sentiment analysis of tweets around COVID-19 vaccination in the US and UK and found that the Twitter sentiment correlated with findings from national surveys [51]. In politics, the sentiment and location of tweets during the 2016 elections in the U.S. and 2017 elections in the U.K., aligned with election results [52]. Additionally, studies have looked at how the sentiment and public discourse shifts in relation to social and current events, with the reaction to the killings of George Floyd, Breonna Taylor, and Ahmaud Arbery [18] and increase in anti-Asian sentiment during the beginning of COVID-19 pandemic [19]. In the wake of the overturning of Roe v Wade, researchers additionally monitored changes in Google Trends and found that search terms for various contraceptives methods for men (e.g., vasectomy) and women (e.g., tubal ligation; “morning after pill”) spike notably [53].

Although the topic of abortion is often discussed from the framework of ‘personal choice,’ religious conviction, or political leanings, it is important to continue to bear in mind that abortion is a medical procedure, thus making it also a healthcare matter. Studies have shown that banning abortions does not deter women from getting them, it merely increases unsafe abortions [47,54]. Moreover, the general consensus in the medical and scientific community maintains that abortion is safe and endorses safe access to it [54,55]. Studies such as this provide insight into public sentiment around abortion and other health-related topics. It enables policymakers and healthcare providers to identify areas that could benefit from further attention from the medical community, identify and combat misinformation and disinformation, and inform future policies that could affect public health.

### 4.2. Study Limitations

This study also has some limitations. First, our study of Twitter sentiments may represent a segment of society that may not accurately represent the actual sentiments of the entire American citizenry. Like other research assessing public opinion, social desirability bias [56] may have affected how people publicly post. In contexts where they believe there is greater support for their viewpoints, they might be more assertive. However, if they believe they hold views in contradiction to the social norms of an area, they may be more hesitant to publicly post. Additionally, we are unaware how much of the data collected were tweets made by “bots” on Twitter, which are software that autonomously perform actions such as tweeting, retweeting, and liking tweets. We are, therefore, unable to differentiate between tweet hubs with a large number of likes generated by authentic users or by bots. Furthermore, Twitter users differ from the entire U.S. population in important ways; they are typically younger, more educated, wealthier, and identify as Democrat [57]. This study also does not consider other unrelated but nationally trending events that may have occurred during the period of study and may have affected the level of engagement captured. For example, incidents like the Uvalde school shooting which occurred during the event period on 24 May could have influenced a shift away from abortion-related topics within the Twitter sphere. Additionally, a larger research question remains about whether public policy is determined by public sentiment or if public sentiment is shaped by the policies. Examining a larger period covering pre and post Dobbs v Jackson would help answer this question.

Because Twitter does not collect demographic data on its users, our analyses could not investigate whether views differ by, for example, education, race/ethnicity, and age. However, previous research has identified large differences in abortion views. For instance, youngest adults, with some college education or higher, and Asians and Blacks are more likely to disapprove the Supreme Court’s decisions on abortion. Religion also strongly affects abortion views with 71% of white evangelical protestants approving the court’s decision. Conversely, only 47% white protestants who are not evangelical approve the court’s decision [58]. Previous studies have found religion to be among the strongest of all social predictors of abortion attitudes [40]. Further examination of how viewpoints by subgroup as well as national patterns are warranted.

## 5. Conclusions

Abortion draws a strong ideological line between “pro-life”, those who oppose abortion, and “pro-choice”, those who support a woman’s right to access abortion. The debate around abortion is often overtaken by political, ideological, and religious realms but it is important to note that the subject is also a matter of health and healthcare care. Our study leverages Twitter to gauge the public reaction to the overturn of Roe v Wade. Following the leaked SCOTUS opinion and official overturning of Roe v Wade, we saw a large increase in Twitter conversations regarding abortion, women as well as family planning, which may signal increased interest among patients seeking counseling on contraceptives and birth control methods and concern about abortion access. Regional differences were detected in the quantity of Twitter conversations. In some states with trigger abortion bans such as Wyoming, North Dakota, South Dakota, Texas, Louisiana, and Mississippi saw relatively few public Twitter conversations about the reversal of Roe v Wade. Sentiment analysis found more negative sentiment in 2022 compared to 2021 across Roe v Wade topic categories. Leveraging Twitter and other social media allows for efficient and wide scale assessment of the public’s reactions to important societal events. The use of social media for health research is emerging and further elaborations are needed.

## Figures and Tables

**Table 1:** Example tweets across topic categories.

Keyword	Example Tweets
Pro-life	PRAISE OUR LORD JESUS CHRIST THAT THIS UNLAWFUL TRAVESTY HAS BEEN OVERTURNED!!! God answers prayers! #life #ProLife #UnbornLivesMatter #unborn Taking the innocent life of an unborn human is NOT health care. Full stop! By definition that’s murder & NOT a right. The only right we should be talking about here is the right to live. #ProLife If you see conservatives celebrating the leaked draft that suggests #SCOTUS is in favor of overturning #RoeVWade, make sure to thank them for their support of expanding #Welfare in this country. Pro-Life, or, more accurately, Forced Pregnancy, will necessitate it. So, thanks!
Pro-choice	I have never imagined a world in which my daughter would have fewer rights than I did I am repulsed, but unsurprised Your vote for trump did this #Dobbs All of the “pro choice” people that are still in Washington screaming. Time to go home it’s over Just stop already. If you’re pro-choice, you might “identify” as a Christian, but you aren’t one.
Abortion	So women can’t have abortions because of some persons religion. Am I living in a science fiction novel? I’m beginning to wonder. I don’t believe anyone thinks abortion is “good”. I don’t know any women who have been through it, joyously walking in for the procedure.
Family Planning	Telling people they should go get IUD’s and birth control NOW is an oversimplified response. First, IUD’s don’t work for everyone aside from the fact that countless women have testified to the misogynistic and barbaric way they are denied pain relief during placement… I believe a woman should only be obligated to take birth control if sex is planned. What about rape victims? Children that are sexually abused? You going to make them give birth and live the rest of their lives knowing they have a child out there and being forced They already have a plan, you can order a box of pills that are supposed to abort babies, they should be using contraceptives to not get pregnant in the first place
Roe v Wade	A victory overturning Roe and Casey should be strongly celebrated by the pro-life movement, and defined not as a “final victory” but as a “milestone victory”—it marks a significant change & development, not ending the mission but initiating a new chapter to it. The Courts ruling on Roe will have a great impact on the nation by no longer being a nationalized issue. The establishment media cannot be fight in all 50 states at once. The media is lazy & can only focus on one
Women’s rights	YES! Fight for the rights we all deserve—women’s rights, voting rights, worker’s rights, right to affordable healthcare, right to gun safety to ensure life Killing an unborn child isn’t “ reproductive rights”, it’s “ murder rights” killing an unborn baby has ZERO to do with reproduction, it’s everything to do with genecide. The implications of Dobbs extend beyond reproductive choice It will impact the role of big government in other matters of bodily autonomy, getting between doctors and patients Like the right of trans youth and their families to have the right to choose an affirming puberty
Women	Not sure who needs to hear this but there are a lot of uteruses in the supreme court and federal government... Please don’t get distracted by the divisiveness in this media stunt. This is not a man versus woman thing Sad day for #women across the country, especially poor women who don’t have the means to travel out of your state #mybodymychoice What woman terminates a wanted pregnancy? Again, name calling is childish. Making yourself angry at ppl who actually want to fix this is insane. You hate liberals. Therefore nothing they say benefits you. Terrible way to run your life. Limited in scope and thinking. I’ve learned over the years that I have a lot in common with the pro-choice side. We want the same thing for women. We just believe in different methods of getting there. Ever notice how pro life women have a joyous aura while pro choice have a dark cloud that emits from them.

## Data Availability

Data was collected through Twitter API for Academic Research. This API is available to students and scholars affiliated with or employed with academic institutions. Details on eligibility and the application process to get access to this API can be found at https://developer.twitter.com/en/products/twitter-api/academic-research.

## Appendix A

**Table A1:** Keyword List.

Keyword List				
abort	contraception	infanticide	pregnancy	unborn
aborted	contraceptive	intact dilation and extraction	pro choice	vacuum aspiration
abortifacient	contraceptives	jane roe	pro lifers	wade
abortion	curettage	judge alito	pro-choice	womb
abortionist	dilation and curettage	baby killing	pro life	womens rights
abortionists	dilation and evacuation	killing babies	pro-life	women’s rights
abortions	ectopic pregnancy	mifepristone	prochoice	womens’ rights
abortuary	embryo	miscarriage	prolife	woman’s rights
adopt baby	embryos	miscarriages	reproductive rights	woman’s right
alito	contraception	misoprostol	right to life	girl
anti-abortion	family planning	murder babies	roe	girls
antiabortion	fertilization	murdering babies	roe v. wade	woman
body autonomy	fetal	naral	roe vs.	women
bodily autonomy	feticide	naral pro choice	rvw	female
female autonomy	fetus	norma mccorvey	scotus	females
woman’s autonomy	fetuses	partial birth	selective reduction	dobbs
forced birth	forced birth	partial-birth	self-induced abortion	vs jackson
birth control	gemeprost	personhood	sen. sam brownback	v. jackson
forced childbirth	heartbeat bill	plan b	supreme court	v jackson
contraceptive	heartbeat law	planned parenthood	textualist	vasectomy
contraceptives	in utero	pregnancies	trimester	

**Table A2:** Keyword categories.

Categories	Keywords/Key Phrases
pro-life	pro-life, prolife, pro lifers, right to life, antiabortion, anti-abortion
pro-choice	pro-choice, pro choice, prochoice
female, women	female, females, woman, women, girls, girl
abortion	abort, aborted, abortifacient, abortion, abortions
family planning	birth control, contraceptive, contraceptives, plan b, family planning, vasectomy, tubes tied, tubal ligation, vasectomy
women’s rights	body autonomy, bodily autonomy, female autonomy, woman’s autonomy, reproductive rights, women’s rights, women’s rights, women’s rights, woman’s rights, woman’s right

**Table A3:** Example tweets across sentiment categories.

Sentiment	Example Tweets
Neutral	oklahoma democratic women need your support please help support the women of oklahoma for pro choice and affordable and ready access to reproductive healthcare prochoiceoklahoma sendbackup
Sad	the outlook in utah if roe falls is grim there will be very few exceptions for accessing abortion care this means is people with resources will still be able to get care likely out of state and those without resources will be in much more difficult situations without choice
Happy	am pro life for those who have held perfect baby in our arms who was not compatible with life begging hoping pleading praying abortion is murder

**Table A4:** Proportion of tweets in each topic category, by state.

State	Number of Tweets with Keywords	% Pro-Life	% Pro-Choice	% Female, Women	% Abortion	% Birth Control	% Women’s Rights	% Roe v Wade	% Other
Alaska	1179	4.5	3.6	51.9	18.7	3.6	2.6	5.8	26.9
Alabama	5795	3.1	0.9	61.3	16.5	2.7	1.4	5.0	20.2
Arkansas	3181	4.0	1.7	57.5	19.3	3.0	1.4	5.8	19.5
Arizona	12,202	3.3	1.4	54.2	17.7	3.1	2.6	6.5	25.1
California	63,520	3.2	1.6	59.3	16.1	2.7	2.0	6.0	21.7
Colorado	9630	3.8	1.5	53.3	19.2	2.6	2.9	6.4	24.7
Connecticut	4251	3.3	1.3	58.4	14.8	2.8	2.1	7.2	21.8
District of Columbia	8233	4.5	2.5	43.7	21.9	2.3	2.1	8.9	31.7
Delaware	1654	2.7	1.0	65.1	14.1	2.4	1.0	5.1	18.4
Florida	35,606	3.1	1.4	57.9	17.6	3.0	1.7	5.6	22.2
Georgia	20,940	2.7	1.1	67.3	13.4	2.6	1.1	4.6	17.0
Guam	31		3.2	64.5	19.4	9.7		3.2	9.7
Hawaii	1724	3.7	2.0	54.0	15.7	4.2	2.3	7.6	25.0
Iowa	3324	3.9	1.5	54.2	16.0	2.0	2.4	5.1	27.7
Idaho	2125	2.8	1.6	49.6	21.7	4.0	2.5	5.2	28.0
Illinois	16,048	3.2	1.7	58.9	16.5	2.6	2.0	6.2	20.6
Indiana	6886	3.5	1.6	56.6	18.7	2.8	2.1	6.2	21.6
Kansas	3445	4.8	1.9	48.9	22.3	2.5	2.1	6.3	25.4
Kentucky	5633	4.0	1.7	55.5	19.4	4.0	2.3	6.5	22.3
Louisiana	6958	3.3	1.2	65.6	14.6	3.3	1.5	4.5	16.2
Massachusetts	9956	3.4	1.5	55.0	18.3	2.5	2.7	6.9	22.9
Maryland	10,161	3.1	1.5	61.9	15.0	2.2	1.7	5.3	20.4
Maine	2111	4.4	2.0	49.9	19.1	2.9	5.5	5.8	27.0
Michigan	10,097	3.4	1.6	57.1	17.0	3.1	2.1	7.2	21.5
Minnesota	6810	3.8	1.9	51.5	19.0	3.1	2.1	7.5	25.0
Missouri	9705	3.7	1.9	54.4	19.4	2.9	2.2	6.2	23.5
Mississippi	2471	2.5	0.9	60.0	19.1	3.6	0.9	5.3	18.7
Montana	1260	4.7	2.0	51.9	17.7	4.1	3.3	6.8	26.7
North Carolina	13,993	3.0	1.4	59.9	15.9	2.8	1.8	6.1	22.0
North Dakota	520	4.8	2.7	47.9	25.6	3.8	4.4	7.3	22.3
Nebraska	1983	4.0	2.0	53.5	18.5	3.0	2.9	5.5	22.8
New Hampshire	1734	3.1	2.6	47.2	24.6	2.4	2.5	6.5	25.7
New Jersey	10,572	3.1	1.1	58.2	16.4	2.7	1.7	5.5	24.3
New Mexico	3396	4.2	1.4	48.4	19.1	3.4	1.6	5.0	32.2
Nevada	9033	2.8	1.5	61.1	16.0	2.9	5.2	5.4	20.4
New York	38,606	3.2	1.6	59.0	15.8	2.2	1.9	6.1	22.0
Ohio	14,844	3.9	2.2	53.1	19.4	3.0	1.8	6.8	23.2
Oklahoma	5588	4.7	1.9	56.1	18.8	4.0	1.7	5.7	21.7
Oregon	7757	3.8	1.9	49.4	18.9	2.6	3.0	6.6	28.2
Pennsylvania	19,870	3.5	1.4	59.2	16.2	2.2	2.3	5.9	21.8
Puerto Rico	757	6.1	1.2	47.3	26.3	2.6	1.2	4.4	26.3
Rhode Island	1760	2.8	1.9	54.4	21.9	3.0	1.9	7.2	21.0
South Carolina	6286	3.4	1.1	60.3	17.1	2.3	1.4	6.3	19.7
South Dakota	820	2.2	2.0	44.6	30.2	3.4	1.5	6.5	27.1
Tennessee	10,599	4.3	1.3	57.3	18.3	3.1	1.6	6.9	20.1
Texas	45,389	3.5	1.5	62.0	15.5	3.1	1.6	5.1	19.1
Utah	3595	3.8	1.3	52.8	18.3	3.3	1.6	6.1	26.8
Virginia	13,284	3.8	1.7	55.8	17.8	2.3	2.7	6.7	23.4
Virgin Islands	202	2.5	0.5	63.4	9.9	1.5	1.0	8.9	22.8
Vermont	928	2.9	1.9	54.4	19.5	1.9	2.3	5.8	25.2
Washington	11,674	3.5	1.7	51.5	17.9	3.2	2.8	6.9	27.2
Wisconsin	6166	4.5	1.9	52.5	18.5	3.1	2.5	6.2	24.8
West Virginia	1832	3.8	1.5	47.7	26.3	3.1	1.6	7.0	23.1
Wyoming	370	3.0	0.3	54.9	22.7	3.5	1.6	5.9	21.6
